# Endophyte-mediated pest resistance: mechanisms, evidence gaps, and translational opportunities

**DOI:** 10.3389/frmbi.2026.1881416

**Published:** 2026-08-24

**Authors:** Soumik Dey Roy, Debamitra Chatterjee, Ritika Thakur, Swarnali Bhattacharya, Ajoy Kumar Mukhopadhyay, Palash Mondal, Aniruddha Pramanik, Hirak Chatterjee

**Affiliations:** 1 Department of Agriculture, School of Agriculture, Brainware University, Barasat/Kolkata, West Bengal, India; 2 Department of Agricultural Entomology, Palli Siksha Bhavana, Visva-Bharati University, Bolpur, West Bengal, India; 3 Department of Entomology, Chaudhary Sarwan Kumar Himachal Pradesh Krishi Vishvavidyalaya, Palampur, Himachal Pradesh, India; 4 Department of Agricultural Entomology, Faculty of Agriculture, Bidhan Chandra Krishi Viswavidyalaya, Nadia, West Bengal, India

**Keywords:** antibiosis, antixenosis, biocontrol, multiomics integration, tritrophic interactions, volatile organic compounds (VOCs)

## Abstract

Escalating crop losses caused by insect pests, together with pesticide resistance, environmental persistence, and increasing regulatory scrutiny of conventional agrochemicals, have intensified interest in biologically based pest-management strategies. Endophytic bacteria and fungi represent a promising avenue for crop protection because they can influence plant defense, pest performance, and multitrophic interactions within plant tissues. This review critically synthesizes the ‘landscape’ of endophyte-mediated pest resistance—the ‘journey’ of microbes from colonization to field protection—through direct antagonism by insecticidal metabolites and enzymes, immune priming, signaling crosstalk (i.e., jasmonic acid, salicylic acid, and ethylene pathways), volatile organic compound-mediated indirect defense, and microbiome restructuring. We further evaluate the strength of evidence across laboratory, greenhouse, and field studies, distinguishing well-supported mechanisms from responses that remain context-dependent or insufficiently validated under agronomic conditions. This implies that endophyte-based pest resistance will remain difficult to translate unless colonization stability, host specificity, ecological trade-offs, formulation performance, biosafety, and regulatory requirements are addressed together. Future progress will require standardized efficacy testing, multiomics-based mechanism validation, improved delivery systems, predictive strain-selection pipelines, and transparent biosafety frameworks. By linking mechanism, evidence strength, and deployment barriers, this review provides a translational framework for moving endophyte-mediated pest resistance from experimental promise toward field-ready crop protection.

## 1 Introduction

Rising global demand for food—projected to increase by approximately 50% by 2050—combined with annual crop losses of 20–40% to pests and diseases, estimated to cost about US$220 billion, has intensified the search for durable and environmentally compatible crop-protection strategies (Waqar et al., 2023). This need has become more urgent as conventional pesticide-based management faces mounting constraints, including resistance development, pest resurgence, environmental contamination, residue concerns, and tightening regulatory scrutiny (Fite et al., 2023; Tariq et al., 2025). In this context, plant-associated microbial communities, particularly endophytes (i.e., symbionts residing within plant tissues), have attracted considerable attention as biologically embedded partners that can enhance host resistance to insect pests and pathogens (Waqar et al., 2023; Aravinthraju et al., 2024; Hendra et al., 2026).

Endophytes (**Box 1**) can influence plant defense through multiple, often interacting mechanisms. These include direct antagonism through insecticidal metabolites (i.e., compounds exerting direct toxicity) or lytic enzymes, modulation of host immune pathways, production or induction of bioactive secondary metabolites, alteration of plant volatile organic compound profiles, and restructuring of plant-associated microbial communities (Fite et al., 2023; Waqar et al., 2023; Hendra et al., 2026). In several systems, endophyte colonization has been associated with the induction or priming of systemic resistance, altered jasmonic acid/salicylic acid/ethylene signaling, increased production of defensive metabolites, and reduced herbivore feeding, survival, fecundity, or host preference (Pandey et al., 2023; Jain et al., 2024; Serepa-Dlamini et al., 2024). These effects position endophytes as potentially valuable components of sustainable pest-management strategies, particularly where conventional chemical control is ecologically or economically constrained.

Box 1 **What is an Endophyte?**

The term “endophyte,” derived from the Greek *endo* meaning “within” and *phyton* meaning “plant,” was introduced by Anton de Bary in 1866 to describe organisms living inside plant tissues without causing overt disease symptoms, building on earlier observations of plant-associated microbes by Johann Heinrich Friedrich Link in 1809 (Collinge et al., 2019; Kurissery et al., 2019; Lengrand et al., 2024). In contemporary plant–microbe research, endophytes are understood as microorganisms that inhabit internal plant tissues for at least part of their life cycle without producing visible disease symptoms (i.e., they facilitate asymptomatic colonization). They include fungi, bacteria, actinomycetes, archaea, and, less commonly, other microbial associates; in pest-resistance research, the most frequently studied groups include fungal endophytes such as *Beauveria*, *Metarhizium*, and *Epichloë*, as well as bacterial endophytes such as *Bacillus*, *Pseudomonas*, *Streptomyces*, and related plant-associated taxa (Garrido et al., 2025; Liao et al., 2025).

Furthermore, a distinction is required between fungal and bacterial classification frameworks. The clavicipitaceous/nonclavicipitaceous framework applies specifically to fungal endophytes and should not be generalized to all endophytes (Rodriguez et al., 2009). Clavicipitaceous endophytes, particularly *Epichloë* species associated with grasses, are often vertically transmitted and may produce alkaloids that reduce herbivore feeding, survival, or reproduction. Nonclavicipitaceous fungal endophytes are more taxonomically diverse, frequently horizontally acquired, and may colonize roots, stems, leaves, or seeds depending on host genotype, tissue identity, plant developmental stage, and environmental conditions (Chitnis et al., 2020; Fite et al., 2023; Verma et al., 2022).

Bacterial endophytes are more appropriately described according to their origin, transmission route, tissue niche, and functional traits. They may enter plants through roots, wounds, stomata, hydathodes, flowers, or seeds, and their persistence depends on compatibility with host immunity, competition with resident microbiota, and environmental filtering (Anjum et al., 2019; Sena et al., 2024). In pest resistance, endophytes may contribute through antibiosis, antixenosis, induced systemic resistance, altered plant nutrition, secondary metabolite production, volatile signaling, or recruitment of protective microbial communities (Sohrawardy and Islam, 2022; Vandana et al., 2024). Their effects are therefore best interpreted as context-dependent plant–microbe–pest interactions rather than as universally beneficial associations—this suggests that these associations exist within a fluid 'landscape' of shifting ecological outcomes.

However, the practical value of endophyte-mediated resistance depends on more than the demonstration of pest suppression under controlled conditions. Endophyte performance is shaped by host genotype, microbial strain identity, tissue specificity, plant developmental stage, environmental conditions, resident microbiota, and pest feeding guild (Chitnis et al., 2020; Fite et al., 2023; Jain et al., 2024). This suggests that the same endophyte may function as a beneficial symbiont, a weak colonizer, a neutral associate, or even a latent pathogen depending on ecological context. This context dependency is one reason why laboratory and greenhouse success does not always translate into consistent field efficacy.

Furthermore, we still face important mechanistic gaps in this area. The genetic, biochemical, and epigenetic processes that regulate endophyte entry, host recognition, immune evasion, tissue persistence, and defense activation are still incompletely resolved (Chitnis et al., 2020; Jain et al., 2024). Similarly, many studies document altered pest performance without fully distinguishing direct microbial antagonism from plant-mediated defense, growth promotion, volatile-mediated indirect effects, or microbiome restructuring. Resolving these uncertainties is essential for converting endophytes from promising biological associates into reliable crop-protection tools.

We critically synthesize current knowledge of endophyte-mediated pest resistance—the ‘hidden’ struggle for plant defense—with emphasis on mechanism, evidence strength, and translational limitations. It examines direct antagonism, immune priming, hormonal signaling, volatile-mediated indirect defense, microbiome modulation, evidence from crop–pest–endophyte systems, formulation and delivery constraints, biosafety, regulation, and multiomics-enabled discovery. This suggests that we must move beyond treating endophytes as universally beneficial agents; we evaluate where evidence is robust, where it remains ‘context-dependent,’ and what is required to bridge the gap toward field-ready application.

## 2 Review approach and evidence prioritization

We developed this review as a critical narrative synthesis, rather than a formal meta-analysis. Relevant literature was identified from major scholarly databases, including Web of Science, Scopus, PubMed, Google Scholar, and publisher databases, using combinations of the following terms: “endophyte,” “endophytic fungi,” “endophytic bacteria,” “plant–microbe interaction,” “insect pest resistance,” “induced systemic resistance,” “jasmonic acid,” “salicylic acid,” “volatile organic compounds,” “microbial volatiles,” “entomopathogenic fungi,” “microbiome,” “multiomics,” “biocontrol,” and “field efficacy.” Priority was given to peer-reviewed studies that directly linked endophyte colonization with insect performance, plant defense signaling, metabolite production, microbiome shifts, or field-level pest suppression. Studies were classified by the type of evidence they provided (e.g., mechanistic, functional, and applied evidence). This implies that we must distinguish between laboratory-controlled phenomena and field-validated outcomes. Where evidence was limited to controlled conditions, context-dependent, or lacking field validation, these limitations are explicitly noted.

## 3 Conceptual and ecological framework of endophytes relevant to pest resistance

Endophytes (i.e., microbial associates inhabiting internal plant tissues without producing visible disease symptoms) comprise bacteria, fungi, archaea, actinomycetes, and other diverse lineages that rely on the host for at least part of their life cycle (Samal et al., 2023). Their ecological role is best understood along a continuum rather than as a fixed beneficial state. Depending on host genotype, microbial strain identity, environmental conditions, tissue niche, and biotic stress context, an endophyte may act as a mutualist, commensal, opportunist, or latent pathogen (Fite et al., 2023; Dargiri et al., 2024). This functional plasticity is central to pest-resistance research because the same microbial association may generate different outcomes across crops, cultivars, pest guilds, and environments.

Endophyte-mediated pest resistance can arise through direct and indirect mechanisms. Direct effects include insecticidal or antifeedant metabolites, lytic enzymes, toxins, and other bioactive compounds that reduce herbivore survival, development, feeding efficiency, or reproduction (Anjum et al., 2019; Lengrand et al., 2024). Bacterial genera such as *Bacillus* can produce lipopeptides and other defense-associated metabolites, whereas fungal endophytes may synthesize alkaloids, terpenoids, phenolics, or related compounds associated with antibiosis and antixenosis (Bamisile et al., 2021; Parveen et al., 2026). Indirect effects include immune priming, hormonal crosstalk, altered plant nutritional quality, volatile-mediated recruitment of natural enemies, and changes in the resident plant microbiome—this suggests that the endophyte effectively ‘re-engineers’ the plant’s defensive landscape.

The conceptual value of endophytes lies in their ability to link plant physiology, microbial ecology, and pest interactions within a single defensive framework. Unlike externally applied pesticides, endophytes may act from within plant tissues and may influence defense before, during, and after pest attack. However, this embedded lifestyle—their ‘journey of transition’ within the host—creates inherent uncertainty. Effective pest suppression depends on successful colonization, persistence in relevant tissues, expression of defensive traits at the correct developmental stage, and compatibility with the host plant’s immune system and resident microbiota (Chitnis et al., 2020; Xia et al., 2022; Pandey et al., 2023).

Several ecological constraints must therefore be considered before endophytes can be treated as reliable pest-management agents. Horizontally transmitted endophytes may fail to establish due to competition with native microbiota or unfavorable environmental conditions, whereas vertically transmitted endophytes may confer greater persistence but can impose host fitness costs or produce metabolites with non-target risks (Abdelrazek et al., 2020; Chitnis et al., 2020). Similarly, a defense-inducing endophyte may reduce pest pressure but also redirect plant resources away from growth or reproduction. This implies that we must move beyond viewing these as simple two-partner interactions and instead evaluate endophyte function at the level of the plant holobiont.

A rigorous framework for endophyte-mediated pest resistance should therefore ask four linked questions: whether the endophyte colonizes the target host reproducibly; whether colonization changes plant defense, chemistry, or microbiome structure; whether those changes reduce pest performance or damage; and whether the effect persists under realistic agronomic conditions. This framework provides the basis for distinguishing experimentally interesting endophytes from candidates with genuine translational potential.

## 4 Diversity, colonization ecology, and host specificity

The taxonomic and functional diversity of endophytes generates substantial variation in plant–pest outcomes because colonization is shaped by microbial identity, host genotype, tissue specificity, plant developmental stage, and environmental filtering (i.e., the extrinsic factors that limit microbial survival) (Kaur et al., 2023; Kumari et al., 2023). Endophytes may enter plants through root cracks, lateral-root emergence zones, stomata, hydathodes, wounds, flowers, or seeds, after which successful establishment depends on adhesion, movement through intercellular or vascular spaces, tolerance of plant antimicrobial defenses, and compatibility with host immune surveillance (Chitnis et al., 2020; Fadiji and Babalola, 2020; Tripathi et al., 2022; Semenzato and Fani, 2024; Kuźniar et al., 2025). These colonization steps determine whether an introduced strain remains localized, spreads systemically, persists through the crop cycle, or fails to establish under field conditions.

Host specificity is a central determinant of pest-resistance outcomes. Some endophytes form narrow associations with particular host lineages or tissues, whereas others colonize multiple crops but express different functional traits depending on host chemistry and physiology (Saunders et al., 2010; Partida-Martínez and Heil, 2011; Li et al., 2023; Parveen et al., 2026). The same microbial species may therefore produce strong antibiosis (i.e., biochemical growth inhibition) in one host but weak or inconsistent effects in another, because plant metabolites, nutrient availability, and immune status influence microbial growth and secondary metabolism (Li et al., 2023; Parveen et al., 2026). Genetic variability within endophyte populations further affects colonization efficiency, secondary metabolite production, and the long-term stability of beneficial associations (Saikkonen et al., 2020; Garrido et al., 2025).

Furthermore, transmission mode influences persistence and translational potential. Vertically transmitted fungal endophytes in grasses can provide relatively stable protection across plant generations, but their benefits may be constrained by alkaloid-associated toxicity, host fitness costs, or environmental modulation of metabolite expression (Saikkonen et al., 2020; Garrido et al., 2025). Horizontally acquired fungal and bacterial endophytes offer greater flexibility for crop inoculation, but they face stronger barriers from inconsistent colonization, competition with resident microbiota, and sensitivity to soil, climate, and management conditions (Santos et al., 2022; Kumari et al., 2023). Moreover, colonization ecology is not a peripheral issue; it is a prerequisite for translating laboratory bioactivity into reliable pest suppression under agronomic conditions.

Understanding endophyte-mediated pest resistance therefore requires more than cataloging microbial diversity. It requires linking strain identity, entry route, tissue distribution, colonization duration, host developmental stage, defensive metabolite expression, and pest-response outcomes. Multiomics approaches can help connect microbial identity with functional traits (e.g., metabolic output), host transcriptional reprogramming, metabolite production, and community-level interactions, but these tools are most useful when integrated with colonization assays and pest-performance data rather than used as descriptive surveys alone (Ali et al., 2023; Pandey et al., 2023; Lengrand et al., 2024; Sadia et al., 2026). This suggests that a functional and biochemical understanding is essential for optimizing strain performance and designing microbial consortia with realistic prospects for sustainable crop protection (Liu et al., 2017; Ercole et al., 2025).

## 5 Approaches for identifying and localizing endophytes within plant tissues

### 5.1 Defining endophytism: a necessary precondition

A rigorous discussion of endophyte-mediated pest resistance must begin with how endophytes are detected, identified, and localized within plant tissues, since inoculation alone does not constitute proof of endophytism (Ayilara et al., 2023; Chen et al., 2022a). A microorganism should be classified as endophytic only when there is direct evidence that it has colonized internal plant tissues, persisted following stringent surface sterilization, and can be reliably distinguished from epiphytic contaminants, rhizoplane-associated microbes, or transient cells adhering to wounds and cuticular surfaces (Ayilara et al., 2023; Chen et al., 2022a). This distinction carries substantial interpretive weight: reduced herbivore performance, altered host chemistry, or apparent defense induction may be erroneously attributed to internal colonization when the causal organism has never been shown to occupy the endosphere (Ayilara et al., 2023). Accordingly, studies investigating endophyte-mediated pest resistance should transparently report the plant tissue examined, the sterilization protocol employed, the colonization assay used, the timing of assessment relative to inoculation, the taxonomic identification method, and the analytical framework linking colonization status to pest-response outcomes (Ayilara et al., 2023; Chen et al., 2022a; Ondzighi-Assoume et al., 2022; Vijayalakshmi et al., 2022).

### 5.2 Culture-dependent isolation from surface-sterilized tissue

Culture-dependent isolation remains the methodological foundation of endophyte discovery, as it alone yields viable isolates amenable to purification, taxonomic identification, re-inoculation, genomic characterization, and functional bioassay (Ayilara et al., 2023; Chen et al., 2022a). In a standard workflow, symptomless roots, stems, leaves, seeds, or flowers are surface-sterilized using sequential treatments of ethanol, sodium hypochlorite, and sterile water rinses before tissue fragments, macerates, or imprints are plated onto general or selective media (Chen et al., 2022a). Rigorous sterility verification is indispensable and typically includes plating the terminal rinse water, imprinting sterilized tissue directly onto agar, and maintaining uninoculated controls to confirm that recovered colonies originate from the internal endosphere rather than residual surface contamination (Chen et al., 2022a).

Bacterial isolates are conventionally characterized through colony morphology, biochemical profiling, and 16S rRNA gene sequencing. In contrast, fungal isolates are first distinguished by colony and microscopic morphology, followed by confirmation via internal transcribed spacer (ITS) sequencing, supplemented with additional loci when ITS resolution proves insufficient (Ayilara et al., 2023; Chen et al., 2022a). The principal strength of this approach lies in generating living strains suitable for re-colonization experiments, whole-genome sequencing, metabolite screening, and formulation development (Ayilara et al., 2023). Its central limitation, however, is culture bias: slow-growing, obligate, nutritionally fastidious, or strongly host-dependent endophytes are frequently overlooked, so recovered isolates often represent only a narrow fraction of the true internal microbial community (Ayilara et al., 2023).

### 5.3 Culture-independent marker-gene sequencing

Culture-independent marker-gene sequencing offers a complementary, unbiased view of endophytic community composition that circumvents culturability constraints (Chen et al., 2022a). Following rigorous surface sterilization, DNA extracted directly from internal tissues can be subjected to 16S rRNA gene amplicon sequencing to assess bacterial assemblages or ITS amplicon sequencing to assess fungal assemblages (Chen et al., 2022a). This approach is particularly powerful for comparative analyses across host genotypes, tissue types, developmental stages, inoculation treatments, and pest-pressure gradients (Chen et al., 2022a).

Nonetheless, amplicon-based detection carries important caveats that must be acknowledged in any high-standard manuscript. Sequence reads may originate from dead cells, relic extracellular DNA, low-abundance contaminants, or organellar sequences rather than metabolically relevant colonizers, while community profiles remain susceptible to distortion from primer bias, differential DNA extraction efficiency, sequencing depth, and reference-database limitations (Ayilara et al., 2023; Chen et al., 2022a). Marker-gene sequencing can therefore robustly characterize community composition and taxonomic diversity, but it cannot, in isolation, establish viability, spatial localization, or a causal relationship with pest-resistance phenotypes (Ayilara et al., 2023).

### 5.4 Shotgun metagenomics and metatranscriptomics

Shotgun metagenomics extends the resolution of marker-gene approaches by capturing functional gene content, biosynthetic gene clusters, broader taxonomic breadth, and strain-level variation that amplicon sequencing cannot resolve (Ayilara et al., 2023). Applied to surface-sterilized tissues, metagenomic data can reveal traits directly relevant to plant defense, including antimicrobial biosynthesis pathways and phytohormone-modulating genes (Ayilara et al., 2023). Metatranscriptomics further advances this framework by identifying which host and microbial genes are transcriptionally active during colonization, herbivory, or abiotic stress, thereby linking static community composition to dynamic functional states (Ayilara et al., 2023).

These omics-based approaches are particularly valuable when the research objective focuses on the endophyte assemblage as an integrated functional consortium rather than on isolating a single candidate strain (Ayilara et al., 2023). They nevertheless share the fundamental limitations of all sequencing-based methods: an inability to conclusively confirm spatial localization within host tissue and persistent difficulty distinguishing metabolically active colonizers from dormant or transient microbial cells (Ayilara et al., 2023; Chen et al., 2022b).

### 5.5 Fluorescence-based visualization and localization

Fluorescence-based imaging methods provide the most direct spatial evidence for confirming true internal colonization, a capability unmatched by culture- or sequencing-based approaches (Vijayalakshmi et al., 2022). Fluorescence microscopy, confocal laser scanning microscopy, fluorescence *in situ* hybridization (FISH), fluorescent dye staining, and GFP- or other fluorescent-protein-tagged strains enable visualization of endophytes in roots, stems, leaves, seeds, vascular bundles, epidermal entry points, intercellular spaces, and cortical tissue (Ondzighi-Assoume et al., 2022; Vijayalakshmi et al., 2022). These techniques uniquely distinguish superficial attachment from genuine endophytic residency and reveal whether colonization remains localized near the point of entry or disseminates systemically (Ondzighi-Assoume et al., 2022). They are especially valuable for validating colonization pathways following seed treatment, root dipping, foliar application, or soil inoculation (Ondzighi-Assoume et al., 2022).

Interpretive caution is nevertheless warranted. Plant autofluorescence, limited probe penetration into dense or lignified tissue, imperfect probe specificity, transformation-associated fitness costs in tagged strains, and inherently low throughput can all confound results (Vijayalakshmi et al., 2022). Fluorescent tagging itself may alter microbial physiology or competitive fitness relative to the wild-type strain, and microscopic visualization alone does not establish that observed cells are metabolically active or mechanistically responsible for an observed pest-resistance phenotype (Ondzighi-Assoume et al., 2022).

### 5.6 Integrated designs linking colonization to function

The most methodologically robust studies do not rely on any single line of evidence but instead triangulate across complementary approaches. Surface-sterilized tissue isolation establishes that viable microorganisms can be recovered from within the plant; 16S rRNA gene or ITS sequencing confirms taxonomic identity; amplicon sequencing and metagenomics characterize community structure and functional potential; and fluorescence-based imaging verifies tissue-level localization and colonization pattern (Ayilara et al., 2023; Chen et al., 2022a; Ondzighi-Assoume et al., 2022; Vijayalakshmi et al., 2022). Critically, for studies of pest resistance, these identification and localization data must be explicitly linked to functional endpoints such as herbivore survival, feeding behavior, fecundity, host preference, plant tissue damage, defense-gene expression, secondary metabolite accumulation, volatile organic compound emission, or broader microbiome restructuring (Ayilara et al., 2023). This integrative requirement is not a methodological formality but a scientific necessity: it is the only way to distinguish genuine endophyte-mediated resistance from epiphytic artifacts, inoculation-associated confounds, generalized plant stress responses, or spurious correlations between microbial presence and pest performance that lack demonstrated mechanistic support (Ayilara et al., 2023; Chen et al., 2022b).

## 6 Mechanistic basis of endophyte-mediated pest resistance

Endophytes confer pest resistance through a set of direct and indirect mechanisms that differ in evidentiary strength and translational maturity. These mechanisms include direct antagonism by insecticidal metabolites or enzymes, host immune priming, hormonal and transcriptional reprogramming, volatile-mediated indirect defense, and microbiome-level restructuring (Chandraleka et al., 2025). In practice, these mechanisms often interact. A single endophyte may suppress herbivores by combining weak direct toxicity with plant-mediated metabolite accumulation, altered feeding cues, or improved compensatory growth. This implies that mechanistic interpretation requires caution: reduced pest performance alone does not identify the causal pathway unless colonization, plant response, microbial activity, and pest endpoints are measured together (Bhardwaj et al., 2023).

### 6.1 Direct antagonism and insecticidal effects

Direct antagonism is one of the most intuitive mechanisms of endophyte-mediated pest resistance, particularly for entomopathogenic (i.e., insect-killing) fungi such as *Beauveria* and *Metarhizium*. These fungi can infect insects via cuticular penetration, enzymatic degradation of the cuticle, internal proliferation, and toxin production; some strains can also colonize plant tissues endophytically (Samal et al., 2023). When such fungi function as endophytes, pest suppression may arise from direct pathogenicity, feeding deterrence, reduced larval growth, impaired reproduction, or plant-mediated changes in defensive chemistry (Bamisile et al., 2021; Aravinthraju et al., 2024), effectively reshaping the defensive ‘landscape’.

However, endophytic antagonism is not limited to overt fungal infection of the pest. Colonized plants may contain or induce bioactive metabolites such as phenolics, terpenoids, saponins, alkaloids, flavonoids, and other compounds that reduce insect feeding, survival, fecundity, or longevity (Bamisile et al., 2021; Francis et al., 2022; Dargiri et al., 2024; Panwar and Szczepaniec, 2024). Endophytes may also produce lytic enzymes, antimicrobial compounds, or secondary metabolites that suppress pathogens and alter the plant’s internal chemical environment, indirectly affecting herbivore suitability (Latz et al., 2018; Akram et al., 2023; Bhardwaj et al., 2023; Fite et al., 2023). These responses are often described as antibiosis, although the term should be used carefully because the underlying cause may be direct microbial toxicity, plant-derived metabolites, or a combination of both.

A critical issue is that direct antagonism is frequently inferred from pest mortality or reduced performance without fully separating fungal pathogenicity from plant-mediated resistance. For example, reduced larval survival on endophyte-colonized plants may result from ingestion of fungal metabolites, altered host-plant chemistry, immune priming, nutritional changes, or a direct mycotic process. Distinguishing these alternatives requires experiments that confirm colonization, quantify fungal presence in plant tissues, identify candidate metabolites, assess plant defense markers, and evaluate whether pest mortality is accompanied by fungal outgrowth or infection symptoms. This implies that direct toxicity should be framed as a ‘plausible mechanism’ rather than as a demonstrated cause.

Synergistic interactions among endophytes, host plants, and resident microbiota may further influence insecticidal effects (i.e., the capacity to suppress herbivore performance). A strain with modest direct toxicity may become more effective when it induces host alkaloid or flavonoid production, interacts with other beneficial microbes, or alters plant volatile profiles in ways that reduce herbivore establishment (Lebody et al., 2021; Li et al., 2022, 2023). Conversely, strong laboratory toxicity may fail in the field if colonization is weak, metabolite expression is unstable, or native microbial communities exclude the introduced strain. Therefore, direct antagonism should be evaluated within the broader plant–microbe–pest interaction rather than as an isolated microbial trait.

Moreover, the translational value of direct antagonism remains high, providing measurable endpoints—mortality, feeding reduction, developmental delay, fecundity suppression, or behavioral avoidance—that facilitate a ‘comparative landscape’ across candidate strains. However, product development requires evidence that these effects persist beyond controlled bioassays and are reproducible under variable field conditions. Molecular characterization of insecticidal metabolites, lytic enzymes, and biosynthetic pathways will be essential for identifying reliable strains, improving formulations, and developing endophyte-based biopesticides with predictable performance (Samal et al., 2023; Wallis and Sisterson, 2024).

### 6.2 Host immune priming and induced systemic resistance

Endophytic colonization can enhance pest resistance by priming the host plant into a defensive state rather than by maintaining constitutively high levels of defense. In this ‘primed’ state (i.e., a condition of heightened preparedness), basal defense remains relatively low in the absence of attack, but subsequent herbivory or pathogen challenge elicits faster, stronger, or more spatially coordinated responses (Clifton et al., 2018; Chaturvedi, 2022; Yu et al., 2022; Jung et al., 2024; Qin et al., 2016). This suggests that the priming model is central to endophyte-mediated pest resistance, providing a mechanism by which plants can enhance inducible defenses while avoiding the ‘metabolic toll’ associated with the continuous activation of costly defensive pathways (Rashid and Chung, 2017; Kamran et al., 2022).

The signaling architecture (i.e., the regulatory landscape) of induced systemic resistance (ISR) is dominated by jasmonic acid (JA), ethylene (ET), and salicylic acid (SA). However, these pathways do not operate as simple linear modules. In general, JA- and ET-dependent responses are strongly associated with resistance to chewing insects and necrotrophic pathogens, whereas SA-associated responses are more frequently linked with biotrophic pathogens and some piercing–sucking insects (Hasanuzzaman et al., 2020; Kamran et al., 2022; Jung et al., 2024). Moreover, this distinction is not absolute—an illustration of the ‘crosstalk’ complexity inherent in biological systems. Endophyte–plant interactions often generate mixed hormonal signatures in which JA, ET, and SA interact with abscisic acid, gibberellic acid, auxin, and reactive oxygen or nitrogen species to determine the final defense phenotype (Rashid and Chung, 2017; Hasanuzzaman et al., 2020; Xia et al., 2022; Samuel and Dines, 2023; Saadaoui et al., 2024).

At the functional level, endophyte-mediated priming may increase the plant’s capacity to accumulate proteinase inhibitors, oxidative enzymes, phenolics, terpenoids, alkaloids, or other deterrent and toxic metabolites (e.g., secondary chemicals) after pest attack (Xia et al., 2022; Li et al., 2023; Bhardwaj et al., 2023). These induced changes can reduce herbivore survival, feeding efficiency, fecundity, or host preference, depending on the pest feeding guild and the plant tissue colonized by the endophyte (Bamisile et al., 2021; Li et al., 2023; Samal et al., 2023). Nevertheless, the strength of evidence varies across systems. Many studies demonstrate altered pest performance under laboratory or greenhouse conditions, but few integrate endophyte colonization, hormone signaling, defense metabolites, and pest suppression into a single field-validated framework. This distinction is critical because priming inferred only from reduced pest performance remains mechanistically weaker than priming supported by coordinated molecular, biochemical, and ecological evidence.

### 6.3 Hormonal crosstalk and molecular signaling networks

The molecular basis of endophyte-induced resistance begins with host perception of microbial- or damage-associated molecular patterns (i.e., MAMPs and DAMPs), followed by intracellular calcium fluxes, reactive oxygen species production, nitric oxide accumulation, mitogen-activated protein kinase (MAPK) signaling, and transcriptional reprogramming (Vryzas, 2016; Lu et al., 2021; Chaudhary et al., 2022; Yu et al., 2022). These early signaling events provide the bridge—a critical ‘threshold’ of transition—between endophyte recognition and downstream defense activation. Although the precise receptors and elicitors remain unresolved for many endophyte–host combinations, available evidence indicates that beneficial endophytes can modulate pathways typically associated with pattern-triggered immunity while avoiding strong immune activation that would lead to rejection of the symbiont (Chitnis et al., 2020; Chaudhary et al., 2022; Pandey et al., 2023; Jain et al., 2024).

Furthermore, downstream defense regulation (i.e., the integration of hormonal signals) involves several interconnected transcriptional and hormonal nodes. JA-responsive transcription factors such as MYC-family regulators, ET-responsive ERF-type factors, SA-associated NPR1-dependent or NPR1-related signaling components, and WRKY transcription factors represent major regulatory points through which plants integrate microbial colonization and herbivore attack (Hasanuzzaman et al., 2020; Kamran et al., 2022; Yu et al., 2022; Jung et al., 2024). These regulatory modules influence the expression of genes involved in pathogenesis-related proteins, proteinase inhibitors, oxidative enzymes, phenylpropanoid metabolism, terpenoid biosynthesis, and alkaloid production (Xia et al., 2022; Li et al., 2023; Samuel and Dines, 2023). This suggests that the molecular effect of an endophyte should not be interpreted solely as ‘JA activation’ or ‘SA activation’; rather, resistance arises from the precise orchestration of the timing, amplitude, and tissue specificity of interacting signaling pathways.

This complexity also explains why endophyte-mediated resistance is often inconsistent across host–pest systems. SA (i.e., salicylic acid) and JA/ET (i.e., jasmonic acid/ethylene) pathways may act synergistically, additively, or antagonistically, and the outcome depends on the endophyte strain, host genotype, pest feeding mode, colonized tissue, and abiotic environment (Bastias et al., 2017; Latz et al., 2018; Kamran et al., 2022; Saadaoui et al., 2024). Although ISR (i.e., induced systemic resistance) has traditionally been described as largely SA-independent and JA/ET-dependent, recent evidence indicates that SA can also contribute to beneficial microbe-induced resistance in a context-dependent manner (Latz et al., 2018; Rithesh, 2020; Jung et al., 2024). This suggests that future studies should move beyond single-hormone assays and instead combine hormone quantification, gene expression profiling, mutant- or inhibitor-based validation, metabolite analysis, and pest performance assays. This implies that we must prioritize such integration to distinguish true immune priming from generalized stress responses or growth-mediated changes in pest susceptibility (Bhardwaj et al., 2023; Jain et al., 2024).

### 6.4 VOC- and mVOC-mediated indirect defense and tritrophic interactions

**Figure 1:**
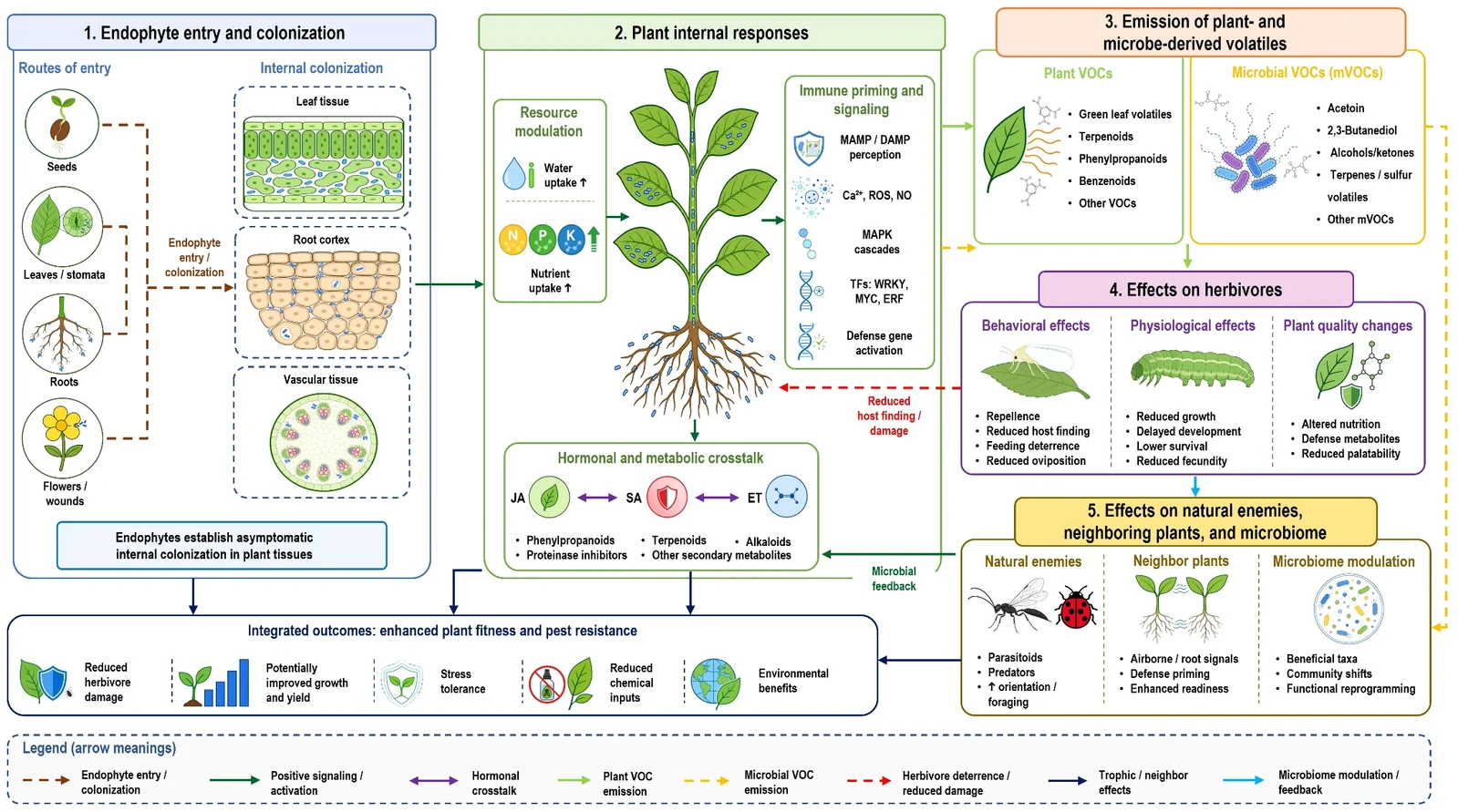
Endophyte-mediated indirect defense and tritrophic communication networks. Root, seed, or foliar colonization by beneficial endophytes can alter plant tolerance, immune priming, volatile signaling, and multitrophic interactions. Internally, endophyte colonization may improve nutrient and water acquisition, while also priming induced systemic resistance through jasmonic acid (JA), salicylic acid (SA), and ethylene (ET)-associated pathways (Rashid and Chung, 2017; Garantonakis et al., 2018; Wei et al., 2020; Harman et al., 2021). These signaling changes can modify plant volatile organic compound (VOC) emission, including green leaf volatiles, terpenoids, phenylpropanoids, benzenoids, and related semiochemicals involved in herbivore deterrence, host-location disruption, predator or parasitoid recruitment, and neighboring-plant priming (Naidoo et al., 2019; González-Mas et al., 2019, 2021; Quesada-Moraga et al., 2022; Zhou and Jander, 2022; Meesters et al., 2024). Microbial VOCs (i.e., alcohols, ketones, terpenes, sulfur-containing compounds, acetoin, and 2,3-butanediol) may further contribute to plant defense priming or insect behavioral modification (Montejano-Ramírez et al., 2024). This suggests that these microbial volatiles act as a ‘chemical shield’ for the host. In the revised figure, all arrows are explicitly labeled to distinguish endophyte entry and internal colonization; nutrient and water uptake/tolerance effects; JA/SA/ET signaling; plant VOC emission; microbial VOC contribution; herbivore deterrence or reduced host finding; natural-enemy recruitment; and neighboring-plant priming.

Endophytes can also influence pest resistance indirectly by altering volatile organic compound (VOC) emission, thereby changing interactions among plants, herbivores, natural enemies, and neighboring plants (Naidoo et al., 2019; **Figure 1**). Herbivore-induced plant volatiles include green leaf volatiles, terpenoids, phenylpropanoids, benzenoids, and other low-molecular-weight compounds that may deter herbivores, interfere with host location, or recruit predators and parasitoids (Zhou and Jander, 2022; Makhlouf et al., 2024). Moreover, in endophyte-colonized plants, these volatile blends may be modified through changes in plant hormone signaling, secondary metabolism, or microbial metabolism itself (Rashid and Chung, 2017; Bamisile et al., 2021; Aravinthraju et al., 2024).

The tritrophic consequences of VOC modulation are especially relevant for biological control. Endophyte-associated changes in volatile blends can increase the attractiveness of herbivore-infested plants to natural enemies while also reducing herbivore host-finding or oviposition (Garantonakis et al., 2018; González-Mas et al., 2019; Quesada-Moraga et al., 2022; Meesters et al., 2024). For example, beneficial microbes can alter JA- and ET-regulated volatile emissions (i.e., key phytohormones), linking immune priming with indirect defense through parasitoid or predator recruitment (Garantonakis et al., 2018; Wei et al., 2020; Harman et al., 2021). Similarly, Bacillus subtilis in tomato has been shown to modify JA-mediated and JA-independent pathways, reducing whitefly host-finding behavior (Ali et al., 2020). This implies that volatile-mediated defense should be interpreted as a ‘dynamic chemical phenotype’—a shifting, complex landscape—rather than as a single-compound response.

Microbial volatile organic compounds (mVOCs) add a further layer of complexity. Bacteria and fungi can emit compounds such as alcohols, ketones, terpenes, sulfur-containing volatiles, pyrazines, acetoin, and 2,3-butanediol, some of which influence plant growth, systemic resistance, pathogen suppression, or insect behavior (Kashyap et al., 2022; Montejano-Ramírez et al., 2024). In plant–endophyte–insect systems, mVOCs may act directly as repellents or attractants, indirectly by priming host defense, or interactively by modifying the plant’s own VOC bouquet (Rashid and Chung, 2017; Montejano-Ramírez et al., 2024). However, evidence for mVOC-mediated pest resistance remains less mature than evidence for general herbivore-induced plant volatiles. Consequently, stronger inference will require experiments that identify volatile compounds chemically and manipulate candidate compounds in synthetic blends; this suggests that we must link these volatile changes to both herbivore behavior and natural-enemy responses under realistic environmental conditions (Johnson et al., 2016; Samal et al., 2023; Zhou and Jander, 2022).

### 6.5 Microbiome modulation and community-level effects

Endophyte-mediated pest resistance should also be considered within the broader plant microbiome, because introduced endophytes rarely act in isolation. They enter tissues already occupied or influenced by resident bacterial, fungal, and archaeal communities. Moreover, their success depends on niche overlap, priority effects, host filtering, microbial competition, and compatibility with the existing microbiome (Christian et al., 2015; Chitnis et al., 2020; Malusà et al., 2021; Sui et al., 2023). This suggests that the community context—the ecological ‘landscape’—explains why strains that perform well in sterile or semi-controlled systems may fail to establish or express defensive traits consistently under field conditions.

Microbiome modulation can contribute to plant protection through several non-exclusive routes. First, introduced endophytes (i.e., symbionts residing within host tissues) may directly alter the abundance or activity of resident microbial taxa, thereby increasing community members associated with nutrient acquisition, pathogen suppression, or defense induction (Christian et al., 2015; Sui et al., 2023; Chang et al., 2021). For example, endophytic colonization by *Beauveria bassiana* in maize has been shown to modify the endophytic bacterial community, increase microbiome network complexity, and enrich potentially beneficial bacterial groups such as *Burkholderia* and *Pseudomonas* in a disease-resistance context (Chang et al., 2021). Although this example concerns plant disease rather than insect suppression, it directly demonstrates that an entomopathogenic fungal endophyte can restructure internal microbial communities, supporting the need to test similar mechanisms in plant–insect systems.

Second, plants under biotic stress may recruit protective microorganisms through “cry for help” processes, in which altered root exudates, volatiles, or defense metabolites favor microbial taxa that improve host resistance (Malusà et al., 2021; Sui et al., 2023). In pest-attacked plants, such recruitment could reinforce resistance by promoting microbes that prime JA/SA/ET pathways (i.e., conserved phytohormone signaling cascades), produce antagonistic metabolites, or improve compensatory growth after damage (Rashid and Chung, 2017; Harman et al., 2021; Pandey et al., 2023). However, direct evidence that endophyte inoculation reshapes the plant microbiome in ways that causally suppress insect pests remains limited. Many studies report pest suppression or changes in plant defense, but fewer combine microbiome profiling, microbial isolation, or synthetic community reconstruction with pest performance assays within a single experimental design.

Third, community-level effects may extend beyond the plant to insects and their symbionts. Herbivores carry bacterial and fungal associates that influence digestion, detoxification, immunity, and vector competence; competition or interference between plant-associated microbes and insect-associated symbionts may therefore alter herbivore performance or pathogen transmission (Vacher et al., 2021). This possibility is mechanistically attractive, yet as a field, we currently have limited information on these dynamics in endophyte-mediated pest resistance. Future studies should treat the system as a linked plant–microbiome–insect–microbiome network, rather than as a pairwise interaction between a single plant and a single introduced endophyte.

A microbiome-centered view also helps define translational constraints—the ‘bottlenecks’ of practical implementation. Effective deployment requires not only selecting strains with insecticidal or defense-priming potential but also predicting whether those strains can establish in the target crop microbiome, persist across plant development, coexist with resident beneficial microbes, and avoid disrupting non-target microbial functions (Qadri et al., 2020; Pandey et al., 2023). Therefore, microbiome modulation should be evaluated for its effects on microbial diversity, taxon abundance, network structure, functional genes, and causal links to pest suppression, rather than inferred from VOC or hormonal responses alone.

## 7 Evidence across crop–pest–endophyte systems

Furthermore, empirical evidence for endophyte-mediated pest resistance remains substantial but uneven across crop–pest–endophyte systems. The strongest support comes from systems in which colonization has been confirmed, pest-performance traits have been measured, and at least one plausible mechanism—such as direct toxicity, antixenosis, altered plant chemistry, immune priming, or volatile-mediated indirect defense—has been evaluated (Castillo Lopez et al., 2014; González-Mas et al., 2021; Quesada-Moraga et al., 2022; María et al., 2025; Saha et al., 2025). In contrast, studies that report only reduced pest abundance without confirming colonization, persistence, or plant-mediated mechanisms provide weaker evidence for endophyte-mediated resistance.

Entomopathogenic fungi (i.e., those capable of infecting insect hosts), particularly *Beauveria bassiana* and *Metarhizium anisopliae*, remain the best-developed examples because they combine insect-pathogenic capacity with endophytic colonization in several crops (Bamisile et al., 2021; Mwamburi, 2021; Aravinthraju et al., 2024). In these systems, pest suppression may result from direct fungal pathogenicity, sublethal effects on larval growth and reproduction, altered feeding behavior, or plant-mediated changes in defense chemistry (Castillo Lopez et al., 2014; González-Mas et al., 2021; Quesada-Moraga et al., 2022; María et al., 2025). However, performance varies with inoculation method, host genotype, colonized tissue, fungal strain, pest feeding guild, and environmental context, which limits direct extrapolation from laboratory or greenhouse assays to open-field deployment (Santos et al., 2022; Kumari et al., 2023; Fite et al., 2023). This suggests that the ‘field gap’—the barrier between controlled conditions and complex ecosystems—remains a significant hurdle.

In contrast, systemic grass endophytes provide a distinct ‘landscape’ of evidence. In *Epichloë*–grass associations, vertically transmitted fungal endophytes can produce alkaloids such as peramine, lolines, ergot alkaloids, and indole-diterpenes that reduce herbivore feeding, survival, or reproduction (Li et al., 2014; Malinowski and Belesky, 2019; Kaur, 2020; Fuchs et al., 2020; von Cräutlein et al., 2021; Sena et al., 2024). These systems offer relatively strong evidence for chemically mediated antibiosis and antixenosis. However, their direct transferability to annual crop systems is limited because many crop-associated bacterial and fungal endophytes are horizontally acquired and less stable across plant generations (Saikkonen et al., 2020; Fite et al., 2023; Garrido et al., 2025).

Furthermore, bacterial endophytes add a ‘tapestry’ of functional diversity. Genera such as *Bacillus* may suppress pests indirectly by priming JA-, SA-, and ET-associated defense pathways, altering host-finding cues, producing antimicrobial or insect-active metabolites, and improving plant vigor under stress (Ali et al., 2020; Waqar et al., 2023; Vandana et al., 2024). These effects are promising but require careful interpretation because improved plant growth, altered nutrition, and induced defense can produce overlapping pest-response phenotypes. Therefore, evidence is most convincing when bacterial colonization, hormone signaling, metabolite profiles, pest behavior, and pest fitness are evaluated together.

To clarify the translational weight of the available literature, we have structured **Table 1** to distinguish experimental scale, colonization method, duration of protection or assessment, reported pest-suppression magnitude, study limitations, and evidence confidence. Evidence confidence was classified as high when colonization, pest suppression, and field or semi-field validation were reported; moderate when colonization and pest-performance effects were shown mainly under laboratory or greenhouse conditions; and low when the evidence remained primarily mechanistic, descriptive, or insufficiently validated against pest outcomes. This classification is not intended to rank study quality, but to identify how close each system is to practical deployment.

**Table 1:** Evidence base for endophyte-mediated defense against insect herbivores across crop systems.

Endophyte/group	Host and target	Colonization/delivery	Assessment window	Quantified endpoint/magnitude	Key limitation	Evidence	Reference
*Beauveria bassiana* and *Metarhizium anisopliae*	Tomato/*Spodoptera frugiperda*	Seedling roots dipped for 2 min in 10^8 spores ml^-1; colonization confirmed by qPCR.	Larvae introduced 14 days after inoculation; larval growth and development assessed during feeding assay.	Fungal DNA detected in >65% of tested plant samples; inoculated plants significantly reduced larval weight gain and slowed development.	Greenhouse study; protection varied with fungal strain and tomato variety; open-field persistence not tested.	Moderate	Mwamburi, 2021
*Beauveria bassiana* UHSB-END1	Tomato/*Spodoptera litura*	Seed treatment, foliar spray, seedling root dip, and combined inoculation approaches.	Colonization assessed at 14, 40, 60, 80, and 120 days post-inoculation; larval mortality monitored up to 15 days.	Colonization reached 100% in leaves, stems, and roots at 14–80 days and remained below 80% at 120 days. *In vivo* mortality exceeded 50% after 7 days; pooled mortality reached 71.33% in the most effective treatment; in planta seed treatment produced 68.33% pooled mortality, and mortality reached 77.50% by 15 days. Pupation was reduced to 10% in some treatments and adult emergence was absent in the strongest treatments.	Controlled conditions; low mycosis suggests mortality may be partly plant/endophyte-metabolite mediated; field validation required.	Moderate-high	Jamunarani et al., 2022
Fungal endophytes including *B. bassiana*, *Hypocrea lixii*, and *Trichoderma asperellum*	Tomato and nightshade/*Tuta absoluta*	Fungal isolates screened for endophytic colonization of roots, stems, and leaves.	Colonization evaluated 4–5 weeks after inoculation; pest life-history parameters assessed across adult and progeny stages.	Twelve of 15 isolates colonized both hosts. *B. bassiana* ICIPE 706 colonized tomato roots, stems, and leaves at 60%, 40%, and 15%, and nightshade roots, stems, and leaves at 70%, 35%, and 15%. Adult survival was reduced to 28.21-32.69% in strongest treatments compared with 52.28% in controls at 5 days; F1 survival at 5 days was reduced to 15.6-24% in tomato and 17-29% in nightshade, compared with 58.16% and 51.23% in controls.	Strong isolate- and host-dependence; screening study requiring formulation and field validation.	Moderate	Agbessenou et al., 2020
*Beauveria bassiana*	Tomato/*Bemisia tabaci*	Foliar spray, root irrigation, and seed dressing; colonization confirmed by nested PCR.	Colonization tracked over time; feeding, oviposition, and Y-tube preference assessed in short-term assays.	Foliar spraying produced 100% colonization within 14 days. At 1 x 10^8 conidia ml^-1, foliar treatment yielded 8.5 +/- 2.02 colonies per 2 g leaf tissue. Egg numbers were reduced to 7.25 +/- 3.49, 45.5 +/- 12.35, and 16.25 +/- 10.47 under selected fungal treatments compared with 33.25 +/- 6.49, 94.75 +/- 12.23, and 75.00 +/- 18.47 in controls. In Y-tube assays, 79.2% of whiteflies chose uninoculated leaves and 20.8% chose treated leaves.	Greenhouse and behavioral evidence; colonization strongly influenced by inoculation method; seed-dressing colonization declined to zero over time.	Moderate	Wei et al., 2020
*Beauveria bassiana*	Melon/*Aphis gossypii* and natural enemies	Endophytic colonization of melon plants, followed by aphid and natural-enemy assays.	Tritrophic assays conducted after colonization.	All inoculated leaves were colonized. Sprayed leaves had 93.33 +/- 3.10 colonies per leaf fragment, and non-sprayed leaves had 24.00 +/- 9.81 colonies per fragment. *Chrysoperla carnea* significantly preferred aphids reared on colonized plants (chi^2 = 9.29, p < 0.01), while predator consumption time and *Aphidius colemani* parasitism, development time, and sex ratio were not significantly altered.	Demonstrates tritrophic modulation rather than direct aphid-population suppression; field-level natural-enemy consequences remain uncertain.	Moderate	González-Mas et al., 2019
*Beauveria bassiana*	Cotton and melon/sap-sucking and chewing herbivores	Endophytic colonization followed by herbivore challenge and VOC profiling.	VOC emissions compared in colonized and non-colonized plants, with and without herbivory.	Endophytic colonization changed plant volatile profiles in a host- and herbivore-dependent manner. VOC markers included benzaldehyde, 4-methyl-octane, (E)-hex-2-en-1-ol, alpha-caryophyllene, decanal, caryophyllene, and 2-ethylhexyl nonyl sulfite depending on host and herbivore context.	Chemical-ecology endpoint; supports VOC-mediated indirect defense but not direct pest-population suppression.	Moderate for VOC modulation; low-moderate for applied pest suppression	González-Mas et al., 2021
*B. bassiana* and *M. anisopliae*	Cucumber/*Aphis gossypii*	Cucumber seeds submerged in conidial suspension; seed colonization and penetration observed by scanning electron microscopy.	Systemic colonization evaluated 28 days after inoculation; aphids assessed after 5 days; plant-growth effects assessed 7 weeks after inoculation.	*M. anisopliae* was recovered from roots, stems, and leaves at 100%, 50%, and 25%; *B. bassiana* was recovered mainly from roots. Aphid numbers were reduced from approximately 230 aphids per plant in controls to about 150–160 aphids per plant in fungal treatments. Total phenolics increased from 94.5 micrograms g^-1 in controls to 101.3 micrograms g^-1 with *M. anisopliae* and 181.8 micrograms g^-1 with *B. bassiana*.	Controlled/non-sterile substrate study; aphid suppression and growth promotion require field validation.	Moderate	Shaalan et al., 2021
*B. bassiana* and *Purpureocillium lilacinum*	Cotton/*Aphis gossypii*	Cotton seeds treated with fungal inoculum; colonization confirmed by culture and PCR.	Greenhouse assays at 7 and 14 days; field trials over two seasons.	In direct pathogenicity assays, *P. lilacinum* and *B. bassiana* caused 60% and 57% aphid mortality over 7 days compared with 10% in controls. Endophytic *B. bassiana* was recovered from 35-55% of sampled tissues and significantly reduced aphid reproduction or abundance in greenhouse and field assays, including field effects in 2012 (p = 0.006) and 2013 (p = 0.016).	Older but strong study; *P. lilacinum* field effects were weaker than *B. bassiana*; colonization detected up to 34 days after seed treatment.	High	Castillo Lopez et al., 2014
*Epichloë coenophiala*, *E. uncinatum*, and related *Epichloë* spp.	Tall fescue and meadow fescue/aphids and other grass herbivores	Primarily vertically transmitted through infected host seed.	Persistent systemic associations across host growth and often across plant generations.	Constitutive alkaloid-mediated antibiosis and antixenosis; lolines, peramine, ergot alkaloids, and indole-diterpenes associated with reduced herbivore feeding, survival, or reproduction.	Strongest evidence from perennial grasses; alkaloid expression is chemotype-, genotype-, and environment-dependent; vertebrate toxicity can constrain deployment.	High for grass systems; limited transferability to annual crops	Kaur, 2020; Fuchs et al., 2020; Sena et al., 2024
*Epichloë festucae* and related *Epichloë* spp.	Fescue grasses and perennial ryegrass/generalist herbivores and Argentine stem weevil	Seedborne systemic fungal association.	Persistent association in naturally infected or selected grass-endophyte combinations.	Alkaloid-mediated antifeedant and toxic effects involving peramine, ergot alkaloids, and indole-diterpenes; protection depends on host-endophyte genotype and alkaloid chemotype.	Translational relevance strongest in pasture grasses; pest-protective alkaloids may be accompanied by livestock-safety or environmental-stability concerns.	High for pasture systems	Malinowski and Belesky, 2019; von Cräutlein et al., 2021
*Epichloë lolii*/*Neotyphodium lolii*	Ryegrass/aphids	Vertically transmitted systemic grass endophyte.	Persistent seedborne association.	Alkaloid-mediated antifeedant effects reduce phloem-feeding performance and host suitability for aphids.	System-specific evidence; direct transfer to non-grass annual crop systems is limited.	High for ryegrass systems	Li et al., 2014
*Bacillus subtilis* and related beneficial bacteria	Tomato and other crops/whiteflies and other herbivores	Bacterial inoculation through seed, root, or rhizosphere-associated delivery depending on study design.	Usually short-term greenhouse or controlled assays following establishment.	Evidence supports altered JA-mediated and JA-independent defense signaling, reduced host-finding cues, and modified plant resistance traits; quantitative estimates of pest suppression should be drawn from primary studies rather than generalized review sources.	Several reports are mechanistic or at the synthesis level; future tables should include only primary bacterial-endophyte studies with confirmed colonization and quantified pest suppression.	Low-moderate unless supported by primary colonization and pest-performance data	Ali et al., 2020; Kashyap et al., 2022; Waqar et al., 2023; Vandana et al., 2024

Endophyte associations are grouped by functional type to clarify their principal mechanisms, experimental scale, and translational readiness. Defense mechanisms include antibiosis, antixenosis, induced systemic resistance (ISR), and indirect defense mediated through volatile organic compounds (VOCs) or tritrophic interactions. Evidence confidence was classified as high when colonization, pest-response outcomes, and field or semi-field validation were reported; moderate when colonization and pest-performance effects were demonstrated mainly under laboratory or greenhouse conditions; and low to moderate when evidence was primarily mechanistic or indirect and lacked field-level validation.

## 8 Greenhouse-to-field translation: performance, delivery, and formulation constraints

The translational journey of endophyte-mediated pest resistance (i.e., defense mechanisms conferred by beneficial microbes residing within plant tissues) from controlled experimental environments to effective field application presents a formidable impediment within this research domain. Laboratory and greenhouse studies often demonstrate colonization, pest suppression, or defense induction under favorable conditions. However, field performance is often compromised by fluctuating temperatures, moisture, soil chemistry, ultraviolet exposure, agrochemical residues, host developmental stage, and competition with resident microbiota (Santos et al., 2022; Kumari et al., 2023; Choi et al., 2026; Poppeliers et al., 2023). This observed instability is particularly salient for horizontally acquired endophytes, as their successful deployment necessitates not merely initial entry into plant tissues but also sustained persistence at biologically relevant densities throughout the critical period of pest pressure. This implies that the ‘ecological footprint’ of such endophytes must be both established and maintained.

Fundamentally, a significant translational barrier emerges from the understanding that metabolite expression and defensive function are not fixed, immutable properties inherent to an endophyte strain. In grass–*Epichloë* systems, for example, production of defensive alkaloids such as lolines and ergovaline can be influenced by host genotype, nutrient status, temperature, drought, and herbicide residues, including glyphosate-associated stress (Lea et al., 2014; Fuchs et al., 2020, 2023). This pervasive environmental sensitivity has two significant practical consequences: First, it implies that bioactivity measured precisely under controlled conditions may not reliably predict field efficacy. Second, it suggests that strains selected solely for their potent bioactivity may ultimately fail as viable commercial products if they cannot maintain consistent colonization, metabolite production, or host compatibility across variable agronomic conditions. Thus, an endophyte’s ‘ecological robustness’ is paramount.

Delivery method strongly determines colonization success. Seed treatment is attractive because it permits early colonization, compatibility with existing seed-handling systems, and relatively low application cost; however, it is most suitable for strains capable of surviving seed storage, germination stress, and early rhizosphere competition (Khang et al., 2023; Khan et al., 2023). Root dipping and root drenching can improve early root colonization in transplanted crops, but these approaches are less convenient for direct-seeded systems and may be labor-intensive at scale. Foliar spraying can target aboveground tissues and may be useful for endophytes that enter through stomata, wounds, or leaf surfaces, but colonization may be more transient because of ultraviolet exposure, rainfall wash-off, cuticular barriers, and phyllosphere competition (Khang et al., 2023; Das et al., 2025). Soil inoculation and organic carrier-based delivery may support rhizosphere establishment, yet they do not guarantee internal colonization unless strain entry, root competence, and host compatibility are demonstrated.

Formulation is therefore not a peripheral technical detail but a determinant of biological reliability. Liquid formulations may provide ease of application but often require careful stabilization to preserve viability. Solid carriers such as talc, peat, vermiculite, compost, biochar, alginate beads, starch-based matrices, or clay-based carriers can improve handling, protect cells or spores from desiccation, and support gradual release (Ganeshan et al., 2021; Khan et al., 2023). Encapsulation, microencapsulation, and immobilization technologies (i.e., physical shielding methods) may further buffer endophytes against environmental stress and extend shelf-life. However, they must be evaluated against strain viability, colonization ability, pest-suppression performance, cost, and farmer usability rather than only laboratory survival (Khan et al., 2023; Adeboye et al., 2025). For pest-resistance applications, an acceptable formulation should preserve viable propagules, maintain infectivity or colonization competence, remain compatible with seed treatments or spray equipment, tolerate local storage conditions, and produce reproducible pest-management outcomes.

Single-strain products may be easier to register and standardize. However, they are vulnerable to context-dependent failure if the strain cannot establish in a particular host genotype, soil type, or resident microbiome. Synthetic or semi-synthetic consortia offer an alternative by combining strains with complementary functions, such as colonization competence, growth promotion, ISR induction, metabolite production, and stress tolerance (Khang et al., 2023; Poppeliers et al., 2023; Choi et al., 2026). However, consortia also introduce additional complexity: member strains may compete, lose function during storage, show unstable ratios after application, or produce emergent effects that are difficult to predict. This implies that their successful utilization requires cautious optimization of inter-strain compatibility. Their use should therefore be guided by compatibility testing, functional redundancy, genome-informed safety screening, and validation under realistic field conditions.

Field-ready endophyte products should meet a higher evidentiary threshold than laboratory candidates. At minimum, candidate strains or consortia should show confirmed colonization of the target crop; persistence through the pest-vulnerable growth stage; reproducible suppression of pest performance or damage; compatibility with agronomic practices; acceptable shelf-life under expected storage conditions; absence of phytotoxicity; minimal risk to beneficial arthropods and resident microbiota; and evidence of efficacy across seasons, cultivars, or locations. Without these criteria, endophyte-based pest resistance will remain an ‘evolutionary dead-end’—promising experimentally but unreliable operationally.

## 9 Ecological trade-offs, biosafety, and regulatory constraints

The ecological value of endophytes as pest-resistance tools depends on a balance among defensive benefits, plant costs, and environmental safety. Endophytes may improve resistance by inducing defense pathways, producing bioactive metabolites, altering plant nutritional status, or reshaping microbial communities, but these processes may also redirect resources away from growth, reproduction, or yield (Rani et al., 2022; Rezaee Danesh et al., 2025). Such trade-offs are not inherently unacceptable; they imply that physiological costs are a necessary component of the broader ‘defensive architecture’. The practical question is whether the defensive gain exceeds the growth or yield penalty under field conditions and whether that balance remains stable across environments.

Furthermore, biosafety concerns are particularly important because endophytes are living agents capable of persistence, dispersal, and interaction with non-target organisms. Potential risks include phytotoxicity, opportunistic pathogenicity, production of undesirable secondary metabolites, horizontal gene transfer, disruption of resident microbial communities, and unintended effects on pollinators, predators, parasitoids, decomposers, or soil food webs (Collinge et al., 2019; Rabiey et al., 2019; Sena et al., 2024; Dubey et al., 2025). These risks are not uniform across all endophytes. For example, vertically transmitted grass endophytes (i.e., those passed from parent to offspring) may provide strong herbivore resistance, but some alkaloid-producing associations can generate anti-vertebrate effects that limit their use in pasture systems (Gundel et al., 2013; Rétif et al., 2023). For this reason, the earlier term “symbiotically modified organisms” is better replaced with the more precise phrase “selected host–endophyte associations carrying nontoxic or low-risk endophyte strains,” which refers to grass cultivars deliberately paired with endophytes that retain pest-protective traits while reducing animal-safety concerns (Gundel et al., 2013; Saikkonen et al., 2016; Rétif et al., 2023).

Regulatory approval remains a major bottleneck because endophyte-based products do not fit neatly into a single conventional category. Depending on their claims and composition, they may be treated as microbial pesticides, biofertilizers, biostimulants, biological control agents, or microbial inoculants (i.e., a highly fragmented regulatory landscape), each with different requirements for identity, purity, efficacy, toxicity, environmental fate, and labeling (Chitnis et al., 2020; Ganeshan et al., 2021; dos Reis et al., 2024). A product intended for pest suppression will generally require stronger evidence than a product marketed only for growth promotion. For endophyte-based pest resistance, regulatory dossiers should ideally include strain-level identification, genome-based screening for toxin or pathogenicity-associated traits, contaminant thresholds, shelf-life data, colonization and persistence data, non-target testing, field efficacy, and clear instructions for crop, timing, dose, and application method.

Commercialization is also constrained by production economics. High fermentation costs, batch-to-batch variability, limited shelf-life, poor compatibility with existing agrochemicals, and insufficient farmer awareness can prevent technically promising strains from becoming usable products (Kumari et al., 2022; Nombamba et al., 2024; Adeboye et al., 2025; Sheikh et al., 2025). Moreover, these problems are amplified when products require cold-chain storage, repeated application, crop-specific inoculation protocols, or narrow environmental conditions. Improving cost-effectiveness will require not only strain selection but also scalable fermentation, robust downstream processing, stable formulation, simple delivery, and clear demonstration of economic benefit relative to conventional pest-management options (Yadav and Yadav, 2017; Khan et al., 2023).

Standardized efficacy testing is urgently needed. Many studies differ in inoculation method, colonization assay, pest stage, plant age, sampling tissue, endpoint measurement, and statistical treatment, making cross-study comparison difficult (Tripathi et al., 2022; Fite et al., 2023; Tariq et al., 2025). This suggests that we currently lack a cohesive ‘standard of evidence’ for evaluating these disparate findings. Future evaluations should report colonization frequency and intensity, spatial distribution within plant tissues, persistence over time, pest-density or damage reduction, yield response, non-target outcomes, and environmental conditions during testing. Multi-location and multi-season trials should be prioritized because they reveal whether the endophyte effect is robust or merely a product of compatibility with controlled environments.

Ultimately, biosafety and regulatory rigor should not be viewed as obstacles to innovation but as prerequisites for credibility. Endophyte-based pest resistance—a complex ‘living protection’ system— will gain acceptance only if products are demonstrably effective, ecologically predictable, safe for non-target organisms, and practical for farmers. The next generation of endophyte technologies should therefore integrate mechanism validation, formulation science, ecological risk assessment, and regulatory planning from the earliest stages of product development rather than treating these steps as post-discovery requirements (Kusari and Spiteller, 2012; Bhardwaj et al., 2023; Kumari et al., 2023; Kuźniar et al., 2025).

## 10 Molecular, multi-omics, and biotechnological advances

Molecular and multi-omics approaches have shifted endophyte research from taxonomic description toward mechanism-oriented analysis. Metagenomics and amplicon sequencing define microbial community structure and functional potential; transcriptomics identifies host and microbial genes responsive to colonization or pest attack; proteomics reveals defense-associated enzymes, signaling proteins, and post-transcriptional changes; and metabolomics links these molecular responses to bioactive compounds, volatile organic compounds, alkaloids, phenolics, terpenoids, and other specialized metabolites involved in pest resistance (Fadiji and Babalola, 2020; Bamisile et al., 2021; Chaudhary et al., 2022; Chen et al., 2022b; Ali et al., 2023; Dargiri et al., 2024; Sahoo et al., 2025; **Table 2**). This suggests that we are witnessing a fundamental move toward holistic, mechanism-oriented synthesis.

**Table 2:** Application of omics technologies in identifying mechanisms and translational constraints of endophyte-mediated pest resistance.

Methodological approach	Pathosystem context	Specific mechanistic output or candidate marker	Translational value/main limitation	Reference
Transcriptomics and metabolomics	*Beauveria bassiana*/tomato/*Bemisia tabaci*	Transcriptomic analysis identified 1,739 DEGs (i.e., differentially expressed genes) and 325 differential metabolites, suggesting that resistance is closely coupled to altered alkaloid biosynthesis, tryptophan metabolism, and specialized metabolites (e.g., alpha-solanine).	The evidence indicates a strong mechanistic link between endophytic colonization and host metabolic reprogramming; however, this implies that the observed impairment of whitefly reproduction requires further field validation to be considered conclusive.	Wang et al., 2023
Metabolomics, transcriptomics, and proteomics	Insect-associated bacteria/maize/*Spodoptera frugiperda*	Bacterial endophyte treatment was associated with shifts in maize metabolic profiles (i.e., defense physiology), suggesting an underlying modulation of larval development responses.	Useful for connecting insect-associated microbes with plant defense and herbivore performance; dissertation-level evidence should be interpreted cautiously until expanded through peer-reviewed field studies.	Achre, 2025
Multi-omics synthesis	Endophytes/cereals and grasses/herbivores	Summarized genetic, transcriptional, and metabolic layers involved in microbiota-mediated stress tolerance and endophyte-associated defensive chemistry.	While this provides a framework for identifying candidate pathways, it does not experimentally validate any single pest-resistance mechanism—indicating the need for further functional inquiry.	Sena et al., 2024
Integrative omics of biosynthetic pathways	Plants and associated microbes/herbivore-relevant defensive metabolism	Linked microbial genes and biosynthetic potential with defensive plant metabolites and candidate pathway discovery.	Supports targeted selection of strains with metabolite-production potential; functional pest assays remain necessary.	Singh et al., 2022
Systems-level omics and computational integration	Microbial communities/diverse crops/insects	Integrated heterogeneous omics datasets to model candidate molecular networks associated with growth promotion and biotic protection.	Useful for systems-level hypothesis generation; predictive value depends on standardized datasets and experimental validation.	Dikobe et al., 2023
Proteomics, metabolomics, and ionomics	Endophytes/diverse crops/insects and abiotic stress	Reviewed protein, metabolite, and ion-homeostasis responses associated with endophyte-mediated stress adaptation.	Indicates measurable biomarkers for stress resilience; pest-specific causal links require stronger validation.	Varadharajan et al., 2025
Transcriptomics and metabolomics	General endophytes/multiple plants/biotic stressors	Synthesized transcript and metabolite changes associated with colonization, host recognition, and stress-response modulation.	Helps prioritize candidate genes and metabolites; many studies remain correlative and not pest-specific.	Chen et al., 2022b
Transcriptomics and metabolomics	Rice/brown planthopper	Connected Bph14/Bph15 resistance-gene background, IAA regulation, and defense-gene dynamics with planthopper resistance responses.	Relevant model for integrating host resistance genes with hormone and metabolite profiling; not necessarily endophyte-specific unless microbial components are directly included.	Jing et al., 2024
Transcriptomics	Endophytes/vegetables/insect pests	Identified differential gene-expression patterns associated with systemic resistance, defense metabolites, and stress-response pathways.	Useful for pathway discovery; claims should be tied to specific genes, pest assays, and confirmed colonization.	Singh et al., 2023
Transcriptomics and proteomics	*Azospirillum brasilense*/wheat/insects	Reported upregulation of *nifH*, *sbpA*, and *trpB*, linking nitrogen fixation, nutrient acquisition, and root chemotaxis with improved plant performance.	Supports indirect resistance through vigor and nutrient-mediated pathways; direct insect-resistance effects require clearer separation from growth promotion.	Chaudhary et al., 2022
Metatranscriptomics	*Fusarium oxysporum* Fo47/*Arabidopsis thaliana*/herbivore-relevant host defense context	Characterized transcriptional plasticity that distinguishes beneficial endophytic behavior from pathogenicity-associated expression patterns.	Useful for understanding the mutualist-pathogen continuum; not a complete pest-resistance validation on its own.	Guo et al., 2021
Shotgun metagenomics	Bacterial endophytes/soybean/insects	Resolved endophytic bacterial diversity and functional-gene potential relevant to plant protection and root colonization.	Helps identify candidate protective taxa and genes; reconstruction or inoculation studies are needed to prove causal pest suppression.	Ayilara et al., 2023
Epigenomics, fluxomics, and ionomics	Fungal endophytes/plants/insects	Proposed multi-level analysis of secondary-metabolite regulation, ion balance, and metabolic flux during host recognition and stress response.	Valuable for mechanistic depth; currently more promising as a research frontier than as a field-screening tool.	Jha et al., 2023
Functional omics	Endophytes/general crops/insects	Linked host genetic background with endophyte diversity, pathogen suppression, and plant immune modulation.	Useful for host-compatible strain selection; pest-specific endpoints and field validation remain needed.	Zhao et al., 2024
RNA-seq, proteomics, and metabolomics	Endophyte-like microbes/plants/insect vectors	Identified candidate molecular changes associated with microbe-mediated alteration of virus transmission by insect vectors.	Relevant for vector-borne disease management; mechanism should be described as candidate or partially resolved unless causality is experimentally demonstrated.	Jangra et al., 2024
Proteomics	Entomopathogenic fungi/*Atractylodes lancea*/insect- and stress-relevant defense context	Identified biomarkers associated with photosynthesis, glycolysis, TCA-cycle activity, and sesquiterpenoid flux during endophyte-associated stress resistance.	Provides functional markers of host metabolic adjustment; direct pest suppression should be tested separately.	Samal et al., 2023
Proteomics using iTRAQ and 2D-PAGE-LC-MS/MS	*Beauveria bassiana*, *Lecanicillium* spp./date palm/insects	Detected post-transcriptional and structural protein changes associated with EPF-host molecular interactions.	Helps characterize colonization and host-response proteins; field-level pest-management relevance requires direct bioassays.	Samal et al., 2023
Metabolomics	Plant-associated microbes/vegetables/insect pests	Quantified defense-metabolite changes, including proteinase inhibitors, and linked them with JA-associated systemic responses.	Stronger when metabolite changes are paired with herbivore feeding, survival, or fecundity data.	Singh et al., 2023
Metabolomics	Endophytes/*Camellia oleifera*/pathogens and insects	Combined DEG and metabolite profiling highlighted flavonoid biosynthesis as a major defense-associated pathway.	Supports flavonoids as candidate defensive metabolites; insect-specific causality requires direct pest bioassays.	Chaudhary et al., 2022
UPLC-MS/MS and MALDI-MSI metabolomics	Plant microbes/maize/aphids	Identified 1-nonene and related VOC-mediated tritrophic signaling associated with ladybird recruitment through SA/ABA-linked pathways.	Strong relevance to indirect defense; field-scale reliability of natural-enemy recruitment remains to be tested.	Jing et al., 2024

The table distinguishes direct mechanistic findings from broader synthesis-level contributions. The “Translational value/main limitation” column clarifies how each approach may support pest-resistance deployment and where evidence remains incomplete. ABA, abscisic acid; DEG, differentially expressed gene; EPF, entomopathogenic fungi; IAA, indole-3-acetic acid; ISR, induced systemic resistance; JA, jasmonic acid; SA, salicylic acid; VOCs, volatile organic compounds.

The greatest value of multi-omics lies not in generating large datasets but in integrating colonization, host recognition, defense signaling, metabolite accumulation, and pest-response phenotypes into a single experimental framework. For example, transcriptomic data can identify differentially expressed genes associated with JA/SA/ET signaling (i.e., jasmonic acid, salicylic acid, and ethylene pathways), MAPK cascades, WRKY or MYC-type transcriptional regulation, pathogenesis-related proteins, proteinase inhibitors, and phenylpropanoid or terpenoid biosynthesis (Jain et al., 2024; Mawcha et al., 2024). Proteomic approaches can then test whether these transcriptional changes are reflected in defense-related proteins or enzymes, while metabolomics can determine whether the predicted pathways produce deterrent, toxic, or signaling compounds relevant to herbivore performance (Chen et al., 2022b; Nazir et al., 2024). Such integration is essential because gene expression alone does not prove resistance function, and metabolite accumulation alone does not identify the regulatory pathway that produced it.

Furthermore, recent studies illustrate the mechanistic potential of this approach. In tomato colonized by endophytic *Beauveria bassiana*, integrated transcriptomic and metabolomic analyses linked whitefly resistance to 1,739 differentially expressed genes and 325 differentially abundant metabolites, including changes in alkaloid biosynthesis and tryptophan metabolism associated with impaired *Bemisia tabaci* reproduction (Wang et al., 2023). Similarly, studies integrating transcriptomics, metabolomics, proteomics, metagenomics, and related approaches increasingly point to candidate defense genes, specialized metabolites, microbial functional genes, and regulatory networks that may explain how endophytes modulate plant resistance (Singh et al., 2022; Dikobe et al., 2023; Samal et al., 2023; Sena et al., 2024; Zhao et al., 2024; Varadharajan et al., 2025).

However, many omics studies remain limited by weak linkage to pest-performance endpoints. This implies that a dataset showing altered transcripts, proteins, or metabolites is mechanistically useful only when connected to colonization intensity, tissue localization, pest feeding, survival, fecundity, behavior, or damage reduction. Future studies should therefore combine molecular profiling with functional validation, including gene-silencing or mutant approaches where feasible, hormone or pathway-inhibitor assays, synthetic metabolite or volatile-blend bioassays, microbial re-isolation, and replicated pest-performance tests. This would help distinguish causal mechanisms from correlative stress signatures or growth-related changes in plant susceptibility.

Artificial intelligence, machine learning, and systems biology provide a ‘new landscape’ (i.e., an integrative predictive framework) for interpreting complex endophyte–plant–pest datasets. Predictive models could integrate strain genome features, host genotype, colonization traits, microbiome composition, metabolomic profiles, pest-performance data, soil conditions, and weather variables to identify candidate strains or consortia with higher probability of field success (Dikobe et al., 2023; Pace et al., 2025; Vompe et al., 2026). These tools should be used cautiously, because agricultural microbiome datasets are often small, heterogeneous, and strongly affected by environmental variation. Their immediate value lies in hypothesis generation, feature selection, risk prediction, and decision support rather than replacing experimental validation.

We propose that biotechnological progress should be directed toward a ‘closed validation pipeline’: strain discovery, genome-informed safety screening, colonization profiling, molecular mechanism testing, metabolite identification, pest bioassays, formulation development, and field validation. Furthermore, emerging fields—such as epigenomics, fluxomics, ionomics, and spatial metabolomics (i.e., high-resolution metabolic mapping)—can deepen our understanding of how endophytes alter host physiology; this implies that translational value will depend on whether these tools can identify reliable markers of colonization stability, defensive metabolite production, and field-level pest suppression (Jha et al., 2023; Chen and Tang, 2025).

## 11 Knowledge gaps and research priorities

Despite rapid progress in endophyte research, several knowledge gaps continue to limit the development of reliable endophyte-based pest-resistance technologies. The first major gap concerns host–endophyte recognition. Although studies increasingly describe defense activation (i.e., the systemic upregulation of host immune signaling), metabolite shifts, or altered pest performance after endophyte inoculation, the molecular events that determine whether a microbial strain is accepted as a beneficial endophyte, restricted as a weak colonizer, or rejected as a potential pathogen remain a ‘black box’ of interaction—this implies that the molecular events remain insufficiently resolved (Kumar et al., 2025; Jain et al., 2024; Sahoo et al., 2025). Future work should therefore focus on identifying microbial elicitors, host receptors, immune checkpoints, and transcriptional regulators that control the transition from colonization to functional resistance.

A second gap is the weak linkage between mechanism and field performance. Many laboratory and greenhouse studies show reduced pest survival, feeding, fecundity, or preference. However, the same effects are less consistently demonstrated under field conditions, where soil pH, moisture, temperature, crop genotype, pest pressure, agrochemical exposure, and competition with indigenous microbiota can alter colonization and defensive function (Sena et al., 2024; Kumar et al., 2025; Nguyen et al., 2025; Malusà et al., 2021; Timmusk et al., 2023; Francioli et al., 2025; Panchal et al., 2026; Hnini et al., 2025). This inconsistency does not invalidate endophyte-mediated resistance; rather, this implies that strain screening must be conducted under ecologically realistic conditions and that colonization stability should be treated as a primary selection trait, not as a secondary observation.

A third priority is to standardize evidence reporting. Studies should clearly document endophyte identity, inoculation method, colonized tissues, colonization frequency or intensity, persistence over time, plant developmental stage, pest stage, pest-performance endpoints, defense markers, and environmental conditions. Without these details, we find it difficult to compare results across host–pest–endophyte systems or to determine whether pest suppression arises from direct microbial antagonism, induced plant defense, altered nutrition, volatile-mediated behavior, or microbiome restructuring (Fite et al., 2023; Tariq et al., 2025). This suggests that standardized reporting would also enhance the value of meta-analyses, regulatory dossiers, and product development pipelines.

The fourth gap concerns functional validation of candidate genes, metabolites, and pathways. Multiomics studies can identify differentially expressed genes, proteins, metabolites, and microbial functional traits (i.e., specific physiological activities)—but many remain correlative, often creating a ‘dead-end’ in mechanistic understanding, unless linked to pest outcomes through targeted validation (Kelliher et al., 2023; Jain et al., 2024; Kumar et al., 2025; Sahoo et al., 2025). Priority should be given to studies that combine transcriptomics, proteomics, metabolomics, and microbiome profiling with hormone quantification, pathway inhibition, mutant or gene-silencing approaches, volatile bioassays, and direct pest-performance tests. Linking secondary metabolites to specific biosynthetic pathways remains especially important because many defensive compounds and their regulatory genes are still incompletely characterized (Tsipinana et al., 2023).

A fifth priority is to develop field-ready delivery and formulation systems. Moreover, endophyte research cannot progress through strain discovery alone; candidate strains must be formulated to maintain viability, colonization competence, shelf life, and compatibility with farmers’ practices (Sergaki et al., 2018; Timmusk et al., 2023; Vompe et al., 2026; Panek et al., 2026). Seed coatings, root dips, soil drenches, foliar sprays, encapsulated formulations, carrier-based products, and synthetic consortia should be evaluated not only for microbial survival but also for internal colonization, defensive function, pest suppression, non-target safety, and economic feasibility.

Recent work on natural bioactive compounds provides a useful parallel for endophyte-based pest resistance. Plant extracts, essential oils, microbial metabolites, and related natural products are increasingly being explored as sustainable alternatives to conventional agrochemicals. However, their deployment faces similar challenges of chemical stability, standardization, formulation, scalability, regulatory approval, and field consistency (Ben El Caid et al., 2026). Endophyte-based technologies should therefore be integrated into the broader landscape of bioactive compound and biopesticide development. This suggests that the future of agricultural innovation relies on such integration, particularly where microbial metabolites, VOCs, or endophyte-derived compounds can be formulated directly or used to guide strain selection.

Artificial intelligence, machine learning, and systems biology offer promising tools for prioritizing strains and predicting field performance, but they should complement rather than replace biological validation. Predictive models could integrate a complex landscape of variables—including microbial genome features, host genotype, colonization traits, microbiome composition, metabolite profiles, weather variables, soil properties, and pest-response data—to identify strain–crop–environment combinations with higher probability of success (Dikobe et al., 2023; Pace et al., 2025; Vompe et al., 2026). However, such models will be reliable only if trained on standardized, well-annotated, multi-location datasets. At present, the most realistic role of AI and machine learning is to support feature selection, risk prediction, candidate ranking, and experimental design.

The final priority is biosafety integration. Future studies should assess non-target effects on beneficial insects, soil microbial functions, native endophyte communities, vertebrate safety, toxin-production potential, and horizontal gene-transfer risks early in development rather than after efficacy has already been demonstrated (Collinge et al., 2019; Rabiey et al., 2019; Sena et al., 2024; Dubey et al., 2025). The most credible path forward is an integrated pipeline that links mechanism discovery, colonization ecology, omics-guided validation, formulation science, ecological safety, and multi-season field testing. Only such a pipeline can convert endophyte-mediated pest resistance from a promising biological phenomenon into a reproducible crop-protection technology.

## 12 Conclusion

Endophyte-mediated pest resistance is best understood as a layered defensive system rather than a single biocontrol mechanism. The most consistently supported mechanisms include direct antagonism by entomopathogenic fungi, alkaloid-mediated antibiosis in systemic grass endophytes, and plant-mediated resistance through inducible hormonal and metabolic pathways. VOC-mediated indirect defense (i.e., volatile-induced systemic plant responses), microbiome restructuring, and microbial consortia offer considerable promise. However, their causal roles in pest suppression remain less consistently validated under field conditions.

The major barrier is no longer whether endophytes can influence pest resistance; that is well established in many controlled systems. This implies that the more important question is whether these effects can be made reproducible across crops, cultivars, soils, climates, pest pressures, and management systems. Colonization instability, context-dependent metabolite expression, competition with resident microbiota, formulation constraints, biosafety concerns, and regulatory uncertainty remain the most urgent obstacles to field deployment.

We anticipate that future progress depends on integrating mechanism and application from the beginning. Candidate strains should be evaluated through a ‘pipeline’ (i.e., a multi-stage assessment framework) that combines confirmed colonization, molecular and metabolite validation, pest-performance assays, formulation stability, non-target safety, and multi-season field testing. Multiomics, systems biology, and predictive modeling can accelerate this process, but they must be anchored in biological validation. Moreover, this implies that endophyte-based pest resistance will only attain ‘field-ready’ status (i.e., ready for commercial application) when experimental efficacy, ecological reliability, product stability, and regulatory credibility are demonstrated in concert.
